# The Change in Public Perception and Knowledge Acquisition Methods of Chronic Kidney Disease Among General Population in Okayama Prefecture, Japan

**DOI:** 10.3390/diseases12110268

**Published:** 2024-10-25

**Authors:** Ryoko Umebayashi, Natsumi Matsuoka-Uchiyama, Hitoshi Sugiyama, Kenichi Shikata, Naoki Kashihara, Hirofumi Makino, Jun Wada, Haruhito A. Uchida

**Affiliations:** 1 Department of Nephrology, Rheumatology, Endocrinology and Metabolism, Okayama University Faculty of Medicine, Dentistry and Pharmaceutical Sciences, Okayama 700-8558, Japan; 2 Department of Medicine, Kawasaki Medical School General Medical Center and Department of Medical Care Work, Kawasaki College of Health Professions, Okayama 700-8558, Japan; 3 Okayama University, Okayama 700-8558, Japan; 4 Kawasaki Geriatric Medical Center, Okayama 700-8558, Japan; 5 Department of Chronic Kidney Disease and Cardiovascular Disease, Okayama University Faculty of Medicine, Dentistry and Pharmaceutical Sciences, Okayama 700-8558, Japan

**Keywords:** chronic kidney disease, CKD perceptance, CKD public education programs

## Abstract

CKD public education plays a very important role in effective chronic kidney disease (CKD) countermeasure. We have been conducting CKD public education programs in Okayama Prefecture since 2007. Here, we aimed to examine the actual status of CKD perceptance and changes in CKD perceptance due to these education programs. The study was conducted on individuals who underwent health checkups at 12 medical institutions across five medical regions in Okayama Prefecture between 1 October and 30 November in 2015, 2019, and 2023. The results showed that overall CKD perceptance has improved over time (perceptance of “CKD” 4% to 7%, “chronic kidney disease” 27% to 34%, 2015 vs. 2023). “Chronic kidney disease” was more commonly recognized than “CKD”, and the elderly were more aware of the disease than younger people. The CKD perceptance improved across all age groups. However, the rate of CKD perceptance is still low, especially among young people. Previously, newspapers were the second most common resource of information about CKD after television. However, the Internet has recently replaced newspapers as the second most common source of information, especially among younger people. Understanding of the exact diagnosis of CKD also remains insufficient. It is necessary to continue more effective CKD public education programs through more intelligible terminology and information sources that match the demographics of target population.

## 1. Introduction

Chronic kidney disease (CKD) is a global public health problem due to its increasing prevalence, significant risk of leading to end-stage renal disease (ESRD), and high medical costs [1,2,3]. In Japan, the estimated prevalence of CKD is approximately 20% [4], and the number of chronic dialysis patients reached 2781 per 1 million population in 2022 [5]. However, it has been reported that even among the patients with advanced CKD, up to half of the patients are unaware of their weakened kidneys [6].

The concept of “CKD” was proposed by the American Kidney Foundation in 2002 [7] with the aim to identify individuals who may develop ESRD, regardless of the clinical course, and to provide standardized care. Sharing a common understanding of CKD among health care professionals and the general population can lead to early detection of CKD and medical intervention [8,9]. Therefore, raising CKD perceptance among the general population is one of the important actions to prevent the progression of kidney disease.

In 2002, the National Kidney Foundation’s Kidney Disease Outcomes Quality Initiative (K/DOQI) launched the clinical practice guidelines, and the Japanese government and academic society jointly launched CKD prevention programs by creating guidelines for primary care physicians and began public education programs in 2007. Additionally, to further promote countermeasures against kidney disease in Japan, the “Kidney Disease Control Commission Meeting” was held in December 2017 over the course of four meetings in the Ministry of Health, Labour and Welfare (MHLW) of Japan [10]. The purpose of this commission is to prevent the deterioration of CKD by early detection of CKD at the stage without subjective symptoms, to provide optimal and high-quality treatment, and to maintain and improve the quality of life of all CKD patients. The report of the Kidney Disease Control Commission was issued by the Ministry of Health, Labor and Welfare in 2018. Five essential items were proposed in this plan; “increase of public CKD perceptance”, “establishment of a regional medical cooperation system”, “improvement of the medical treatment level”, “human resource development”, and “promotion of research and development”. A key performance indicator of decreasing the number of new dialysis patients to ≤35,000 by 2028 was also set [10]. Good examples in several cities have been reported [11]. With the 10-year plan halfway through, an interim report was released, and new goals were set for the next five years [12].

In Okayama prefecture, CKD public education programs were also started and have been ongoing since 2007 [13,14,15,16]. These CKD public education programs are expected to improve the perception of CKD among the general population. Although a single-year CKD awareness survey has been conducted in the past [17], the impact of CKD public education programs on CKD perceptance has not been previously investigated.

Therefore, this study aimed to investigate the effectiveness of ongoing CKD public education programs in Okayama Prefecture on CKD perceptance among the general population. We also examined in detail the status of knowledge acquisition regarding CKD.

## 2. Materials and Methods

Surveys were performed on those who underwent health checkups in 12 medical institutions across the 5 medical regions in Okayama prefecture between 1 October and 30 November in 2015, 2019 and 2023, and those who would consent to answering the questionnaire. The questionnaire investigating the perception of CKD consisted of 6 questions, including gender, age, perception of the term “CKD” and “chronic kidney disease”, information source of knowledge acquisition of CKD or chronic kidney disease, question about the components of diagnosis of CKD, and health insurance (Supplemental Table S1).

This study was approved by the Okayama University Institutional Review Board (No 2309-042) and was conducted in accordance with the Declaration of Helsinki Principles. Informed consents were obtained from all participants who agreed to answer the questionnaire.

All collected questionnaires were sent to Okayama University for data analysis. Statistical analysis was performed using the chi-square test, one-way analysis of variance with Holm–Sidak post hoc, or one-way analysis of variance on Ranks with a Dunn’s post hoc as a multiple comparison test where appropriate, using JPM Pro17 (SAS Institute, Inc., Cary, NC, USA) or SigmaPlot v15.0 (Systat Software Inc., San Jose, CA, USA). A *p*-value < 0.05 was considered statistically significant.

## 3. Results

### 3.1. Participant Characteristics

The number of respondents in the survey performed in 2015, 2019 and 2023 was 7022, 6639 and 7780, respectively. The ages of the respondents ranged from teens to 80s, the majority were aged 40–59 years old and 42–47% were males (Table 1). There was no difference in participant characteristics between the three surveys.

### 3.2. Social (or Public) Perception of the Term “CKD” or “Chronic Kidney Disease”

**Figure 1:**
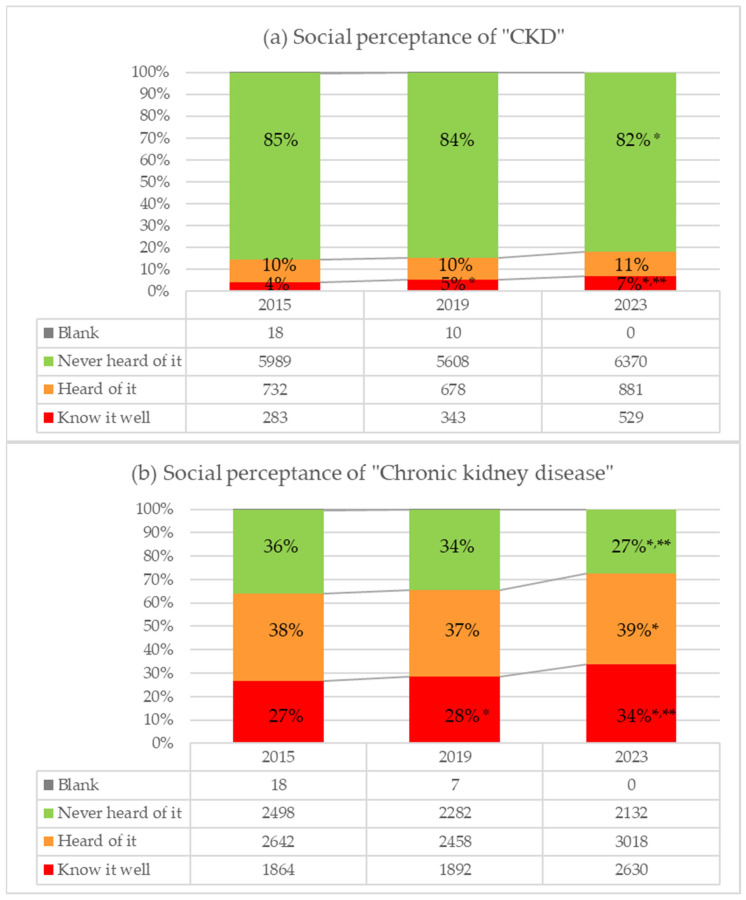
Perceptance of the terms ”CKD” and “chronic kidney disease”. (**a**) Perceptance of “CKD”. (**b**) Perceptance of “Chronic kidney disease”, * shows *p* < 0.05 compared with 2015, ** shows *p* < 0.05 compared with 2019.

The recognition of the term “CKD” gradually increased from 4% to 7% over time from 2015 to 2023; in contrast, the ratio of those who answered “Never heard of it” decreased from 85% to 82% (*p* < 0.0001, Figure 1a). Similarly, the recognition of the term “chronic kidney disease” increased from 27% to 34%, while the percentage of those who were unaware decreased from 36% to 27% (*p* < 0.0001, Figure 1b). Approximately 15% of respondents each year reported that they knew or had heard of the term “CKD”. In contrast, 60–70% of respondents were familiar with or had heard of the term “chronic kidney disease”. There was a clear difference in familiarity with the terms “CKD” and “chronic kidney disease”.

### 3.3. Age-Specific Familiarity with CKD

**Figure 2:**
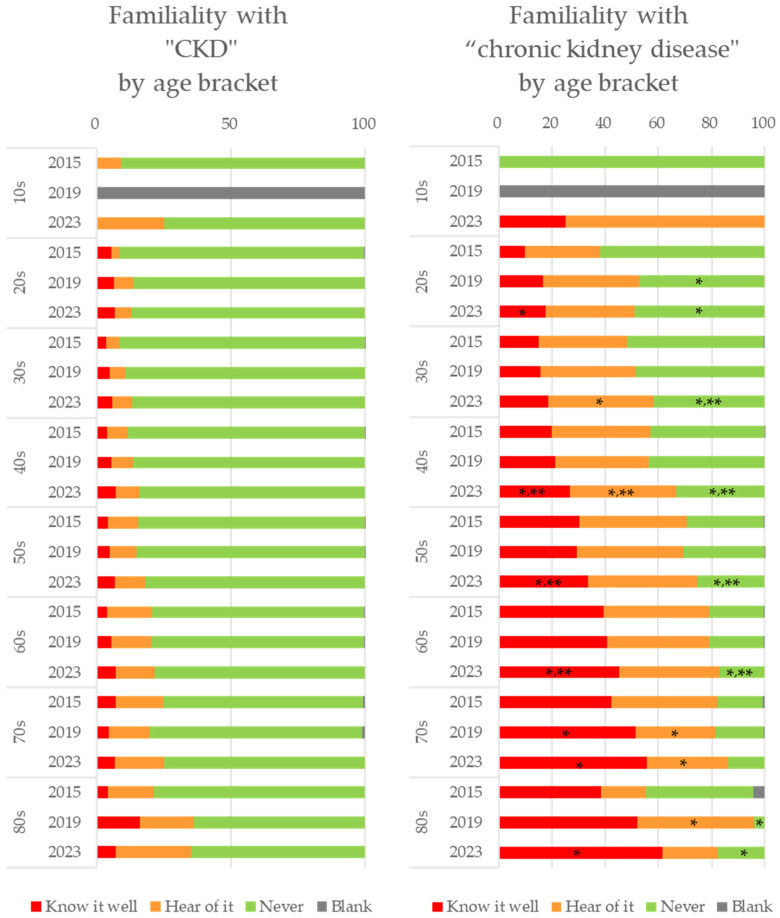
Age-specific familiarity with the term ”CKD” or “chronic kidney disease”, * shows *p* < 0.05 compared with 2015, ** shows *p* < 0.05 compared with 2019.

Regarding age-specific familiarity with the term “CKD” or “chronic kidney disease”, the recognition of both terms increased between 2015 and 2023 in all age brackets (Figure 2). Especially in the ages ranging from 40s to 60s, the recognition of the terms “CKD” and “chronic kidney disease” increased with the statistical significance (* *p* < 0.05 vs. 2015, ** *p* < 0.05 vs. 2019). Moreover, the familiarity with “CKD” and “chronic kidney disease” was particularly high among elder people.

### 3.4. Acquisition Methods of Knowledge on CKD

**Figure 3:**
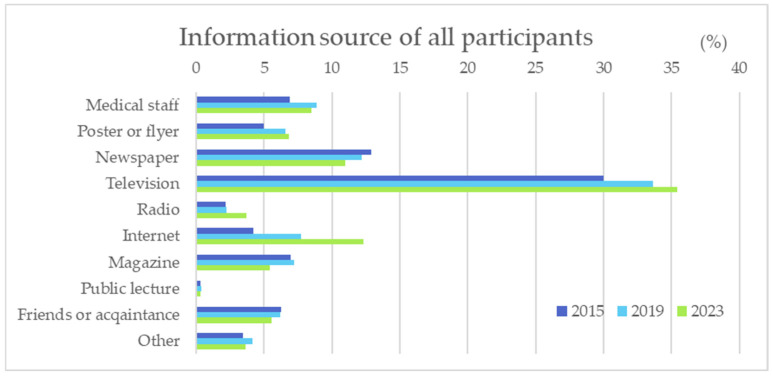
Information source of knowledge acquisition of CKD in all participants.

**Figure 4:**
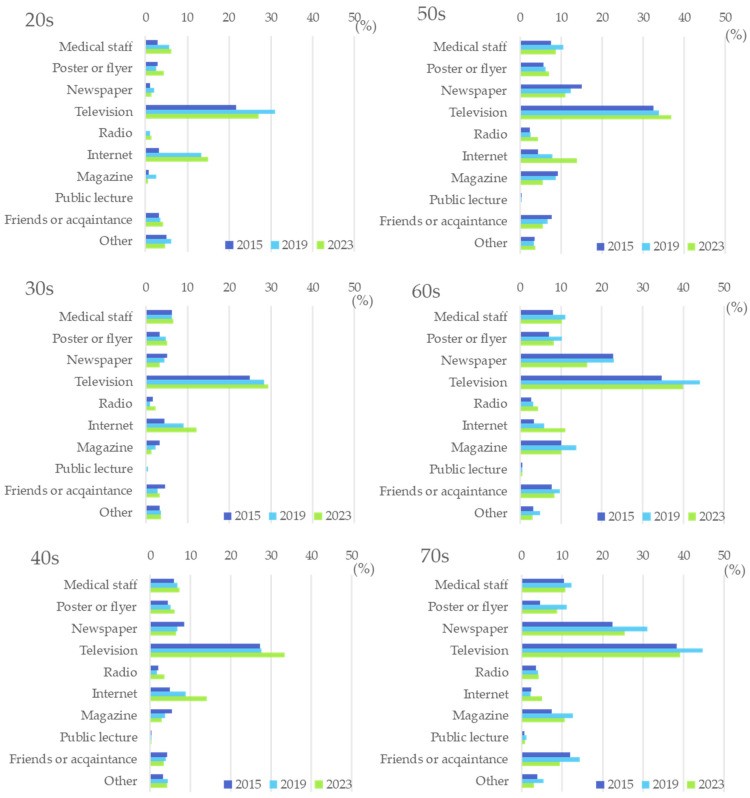
Age-specific information source of knowledge acquisition of CKD.

Television was the top means of learning about “CKD” or “chronic kidney disease” for all respondents, followed by newspapers, the Internet, and medical staff (Figure 3). From 2015 to 2023, the proportion of respondents who acquired knowledge about CKD through “television” and “Internet” increased, while the proportion of respondents who obtained knowledge about CKD through “newspaper” decreased. Regarding age-specific acquisition methods of knowledge on CKD, television was the most common source for all age groups. In the elder age group (50 and older), newspapers were the second common source of information, while the Internet was the second most common method for those in the younger age group (younger than 50) to obtain information (Figure 4).

### 3.5. Understanding of CKD Diagnosis

**Figure 5:**
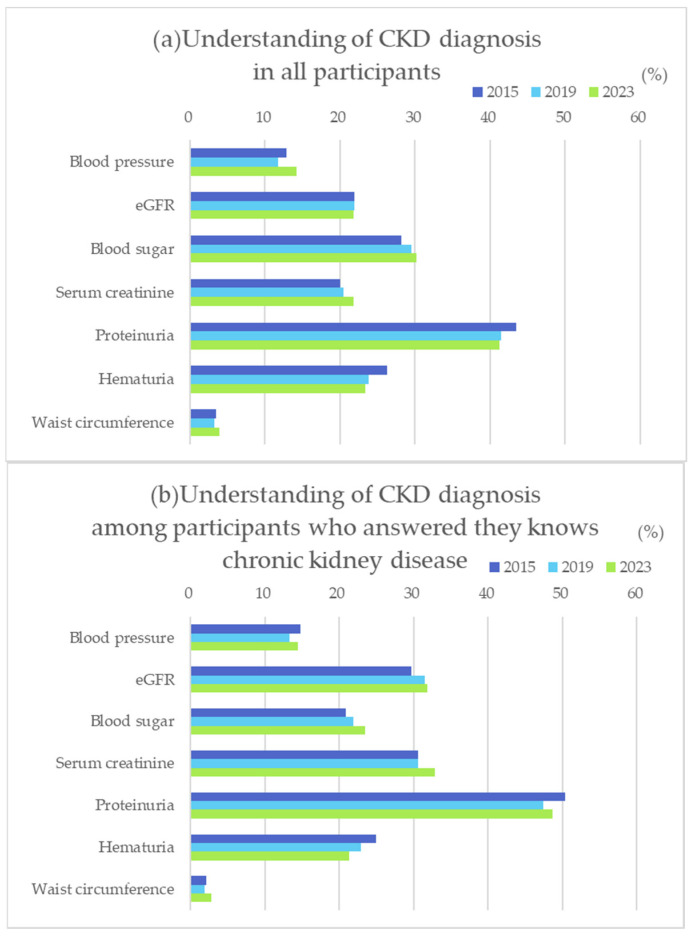
Understanding of CKD diagnosis in all participants (**a**) and in those who answered that they know CKD/Chronic Kidney Disease (**b**).

To further examine the knowledge and understanding about CKD in the public population, we also asked participants about the tests required for the diagnosis of CKD. About forty percent of participants answered that proteinuria was essential for the diagnosis of CKD, while only 20% answered eGFR, or serum creatinine. Conversely, 30% of participants answered blood glucose is necessary, which was the second common answer (Figure 5a). On the other hand, among those who answered that they knew about CKD, nearly 50% answered that proteinuria was necessary to diagnosis CKD, followed by eGFR and serum Cr at 30%, showing a more accurate understanding of CKD than overall participants.

## 4. Discussion

This study evaluated the effectiveness of CKD public education programs that have been ongoing since 2007 in Okayama prefecture, Japan. It is clear that chronic kidney disease has become widely recognized among the general population over the past 16 years, and new findings were obtained regarding the target audience and intervention methods for future CKD public education programs.

In previous reports in Japan, the percentage of those who had no knowledge of CKD was 29% among those who underwent health checkups in 2019 [17], and 36% in an Internet survey of the general population conducted by the Japan Kidney Association in 2022. Although the survey timing and period differed from the above two surveys, the percentage of those who had never heard of CKD in this survey ranged from 27% to 36%, which is almost compatible with these reports. Furthermore, this study examined the transition of perceptance of CKD and found that the term “chronic kidney disease” has gradually become more common in the general population. This study also revealed that the awareness of the two terms “CKD” and “chronic kidney disease” differed greatly, at 7% and 30%, respectively. This discrepancy of recognition between “chronic kidney disease” and its abbreviation “CKD” also has been observed in previous reports [17]. It is likely because “chronic kidney disease” would be easier to understand and more familiar to the Japanese than “CKD”.

In addition, it was found that the older participants were more familiar with CKD. This trend is also observed in the previous two reports in Japan in 2019 and 2022 mentioned above. The reason for this may be that the prevalence of CKD is higher among the elderly [18]. In general, the elderly are more susceptible to various diseases than younger people. People first become concerned about an illness when they themselves become ill. Similarly, in this study, more than 10% of participants aged 70 years and older reported that they had heard about CKD from a medical staff or friend, compared to less than 10% of younger participants. CKD is a familiar problem for the elderly.

Although CKD perceptance was high among the elderly, it was notably low among younger individuals under the age of 40. In Japan, the average age of patients initiating dialysis is 71.4 years old [5]. However, considering the gradual progression of CKD over several to ten years, it is crucial to educate younger populations as well. Research has demonstrated that increasing CKD awareness encourages earlier visits to health care facilities [11,19], facilitating early detection and treatment. Consequently, raising awareness of CKD among younger individuals is essential.

In Japan, the government and academic societies have been promoting CKD public education programs to prevent the onset and worsening of CKD. Especially, Okayama Prefecture has been one of the most advanced in Japan in its efforts to conquer CKD [13,14,15,16]. From early on, the local government, universities, and medical associations have collaborated to implement various initiatives, including public lectures, events, and banner displays related to CKD. The increase in CKD perceptance in 2019 compared to 2015 reflects these ongoing public awareness efforts. Notably, in 2018, the Ministry of Health, Labor, and Welfare (MHLW) released a report by the Kidney Disease Control Commission Meeting, setting the direction for renal disease control for the next 10 years [12]. This report spurred local governments, including Okayama Prefecture, to take measures against CKD. Meanwhile, the COVID-19 pandemic spread worldwide in 2020, reaching Japan as well [20]. Lifestyles changed drastically as people began to minimize contact with others and to stay indoors. As these lifestyles continued, the Internet became a crucial source of information. Beyond everyday uses such as shopping, the rapid adoption of online meetings without direct face-to-face contact accelerated. Additionally, the digital transformation of education and work advanced significantly. Given this social background, the Internet rapidly became an integral part of society in a short period of time since 2020. In general, younger people tend to adopt new cultures more readily than older people. In fact, a survey by Japan’s Ministry of Health, Labor and Welfare found that the internet usage rate for people under the age of 70 was over 90%, while it was low at 67% for those aged 70 to 79 and 36.4% for those aged 80 and over [21]. In this study, we examined sources of information about CKD and found that individuals aged 50 and older primarily obtain their information about CKD from television and newspapers. Meanwhile, television and the Internet were the two leading sources for those younger than 50, especially in 2023. The Internet as a source of information is rising for almost all age groups. However, its percentage is still lower than that of television. The rate of obtaining information from newspapers and magazines is quite low among those under 50 but remains high among those over 50. Therefore, as a better strategy for the promotion of CKD perceptance, providing information through more effective media and methods to different age groups will further increase CKD perceptance in the future.

The survey on understanding CKD diagnosis revealed that while the importance of proteinuria is widely recognized, many participants consider not only eGFR and Cr but also plasma glucose level and blood pressure to be equally important. This may be due to the fact that diabetic nephropathy accounts for approximately 40% of the causes of dialysis initiation in Japan and is the leading cause of new dialysis cases [5]. Additionally, diabetes and metabolic syndrome countermeasures are emphasized in health checkups as crucial for preventing CKD onset and progression. It is also known that treatment goes well when patients diagnosed with CKD have accurate knowledge [22,23]. A survey of the general population conducted in other countries in 2014 also pointed out a lack of knowledge about the diagnosis and symptoms of CKD, and it is considered necessary to increase awareness and provide accurate information about the disease [24,25,26,27]. Continuous CKD education programs for the general population for early detection of the disease are needed in the future.

There are several limitations to this study. This study was conducted only among participants who underwent the health checkup at selected institutions, so that the participants may have relatively high health consciousness. Since this study was conducted on a selected population in a certain area of Japan, it may not accurately reflect the awareness of CKD in the general population.

## 5. Conclusions

While CKD perceptance among the general population has improved in Okayama prefecture due to past CKD public education programs and activity. However, CKD awareness among young people remains notably low, with insufficient understanding. Furthermore, as observed in other Japanese prefectures, young people and middle-aged/elderly individuals utilize different information sources. It is necessary to continue more effective CKD public education programs through more intelligible terms and information sources that match the demographics of the target population.

## Figures and Tables

**Table 1:** Participant characteristics.

Year		2015	2019	2023
Number		7022	6639	7780
Gender	Male	3043 (47%)	2831 (43%)	3271 (42%)
	Female	2917 (44%)	2773 (42%)	4316 (44%)
	Blank	962 (14%)	1035 (16%)	1093 (14%)
Age	10s	11 (0.2%)	0 (0%)	4 (0.1%)
	20s	276 (4%)	223 (3%)	340 (4%)
	30s	942 (13%)	812 (12%)	853 (11%)
	40s	2122 (30%)	1952 (29%)	1977 (25%)
	50s	1974 (28%)	1875 (28%)	2289 (29%)
	60s	1352 (19%)	1314 (20%)	1607 (21%)
	70s	285 (4%)	417 (6%)	622 (8%)
	80s	47 (1%)	25 (0.4%)	57 (1%)
	Blank	13 (0.2%)	21 (0.3%)	31 (0.4%)
Insurance	National Health Insurance		2032 (31%)	2454 (32%)
	Employees’ Insurance		3038 (46%)	3433 (44%)
	Other		1257 (19%)	1397 (18%)
	Blank		312 (5%)	526 (6%)

## Data Availability

The datasets generated during and/or analyzed during the current study are available from the corresponding author on reasonable request. Because Ethics committee approved this protocol only under limited condition of data availability.

## Supplementary Materials

The following are available online at https://www.mdpi.com/article/10.3390/diseases12110268/s1, Table S1: actual study questionnaire.
