# Supplementary material for: The Change in Public Perception and Knowledge Acquisition Methods of Chronic Kidney Disease Among General Population in Okayama Prefecture, Japan
Source: Diseases. 2024 Oct 25;12(11):268. doi: 10.3390/diseases12110268 (PMC11593020; doi:10.3390/diseases12110268)
Supplement: Supplementary file 1 [file diseases-12-00268-s001.zip › diseases-3212027-supplementary.pdf]

Supplementary Table S1. Study questionnaire

**Q1.**	**Please select your gender and age.**
	Male, Female
	20s, 30s, 40s, 50s, 60s, 70s, 80s
**Q2.**	**Are you familiar with the term "CKD"?**
	1) Know it well.
	2) Have heard of it but do not know what it means.
	3) Never heard of it.
**Q3.**	**Are you familiar with the term "chronic kidney disease"?**
	1) Know it well.
	2) Have heard of it but do not know what it means.
	3) Never heard of it.
**If you chose "Never heard of it" in both Q2 and Q3, please go to Q5. Otherwise, please go to Q4.**	
**Q4**	**On what occasions have you heard the term "chronic kidney disease" or "CKD" ? (multiple answers allowed)**
	Poster/flyer, Newspaper, Television, Radio, Inernet, Magazine, Public lecture, Acquaintance, Other
**Q5**	**How is chronic kidney disease (CKD) diagnosed? (multiple answers allowed)**
	Blood pressure, Glomerular filtration rate (GFR), Blood glucose, Serum creatinine, Proteinuria, Hematuria, Waist circumference
**Q6**	**Please select your health insurance.**
	National Health Insurance, Employee's Insurance, Other
